# Augmented quantal release of acetylcholine at the vertebrate neuromuscular junction following tdp-43 depletion

**DOI:** 10.1371/journal.pone.0177005

**Published:** 2017-05-04

**Authors:** Stefania Dzieciolowska, Pierre Drapeau, Gary Alan Barclay Armstrong

**Affiliations:** 1 Départment de Neurosciences and Centre de recherche du centre hospitalier de Université de Montréal (CRCHUM), Montréal, QC, Canada; 2 Integrated Neuroscience Program, McGill University, Montreal, QC, Canada; 3 Department of Neurology and Neurosurgery, the Montreal Neurological Institute, McGill University, Montréal, QC, Canada; EPFL, SWITZERLAND

## Abstract

TAR DNA binding protein (TDP-43) is a 43 kD, predominately nuclear, protein involved in RNA metabolism. Of clinical significance is that the majority of amyotrophic lateral sclerosis (ALS) patients display abnormal accumulation of misfolded TDP-43 in the cytoplasm, which is coincident with a loss of nuclear localization in the afflicted regions of the central nervous system. Little is known about defects that arise in loss-of-function models, in particular synaptic defects that arise at the neuromuscular junction (NMJ). In this report, we examined abnormalities arising at the NMJ following depletion of tdp-43 using a previously characterized mutant *tardbp* (encoding tdp-43) zebrafish line containing a premature stop codon (Y220X) that results in an unstable and degraded protein. Homozygous *tardbp*^Y220X/Y220X^ zebrafish do not produce tdp-43 but develop normally due to expression of an alternative splice variant of *tardbpl* (*tardbp* paralog). Using an antisense morpholino oligonucleotide to knockdown expression of the *tardbpl* in *tardbp*^Y220X/Y220X^ embryos, we examined locomotor defects, NMJ structural abnormalities and release of quantal synaptic vesicles at the NMJ. As in previous reports, larvae depleted of tdp-43 display reduced survival, gross morphological defects and severely impaired locomotor activity. These larvae also displayed an increased number of orphaned pre- and postsynaptic NMJ markers but surprisingly, we observed a significant increase (3.5 times) in the frequency of quantal acetylcholine release at the NMJ in larvae depleted of tdp-43. These results indicate that reduced TDP-43 levels alter quantal vesicle release at the NMJ during vertebrate development and may be relevant for understanding synaptic dysfunction in ALS.

## Introduction

TDP-43 is a 414 amino acid, ubiquitously expressed member of the heterogeneous nuclear ribonucleoprotein (hnRNP) family encoded by the *TARDBP* gene. Structurally, it contains a nuclear import sequence, a nuclear export sequence, two RNA recognition motifs (RRM1 and RRM2), and a c-terminal glycine-rich domain involved in protein-protein interactions [1, 2]. Though its function is not fully known, TDP-43 has been shown to be involved in several steps of RNA metabolism including transcription, splicing and autoregulation of its own cellular expression. Previous work, examining its RNA targets, has established that it is involved in various pathways including synapse formation, regulation of neurotransmitter signaling and neuronal development [3–5]. Interest in TDP-43 stems from the pathological observations that TDP-43 is a major component of cytoplasmic aggregates in ubiquitin-positive tau-negative neuronal inclusions and clearance of nuclear TDP-43 in the majority (>90%) of ALS cases [6]. A recent mouse model expressing inducible human TDP-43 with a defective nuclear localization sequence has locomotor impairment, muscle denervation and motor neuron death which occurred with the accumulation of insoluble cytoplasmic TDP-43 and loss of endogenous mouse Tdp-43 nuclear localization [7]. The synaptic defects that arise following loss of nuclear TDP-43 remain to be elucidated.

**Fig 1:**
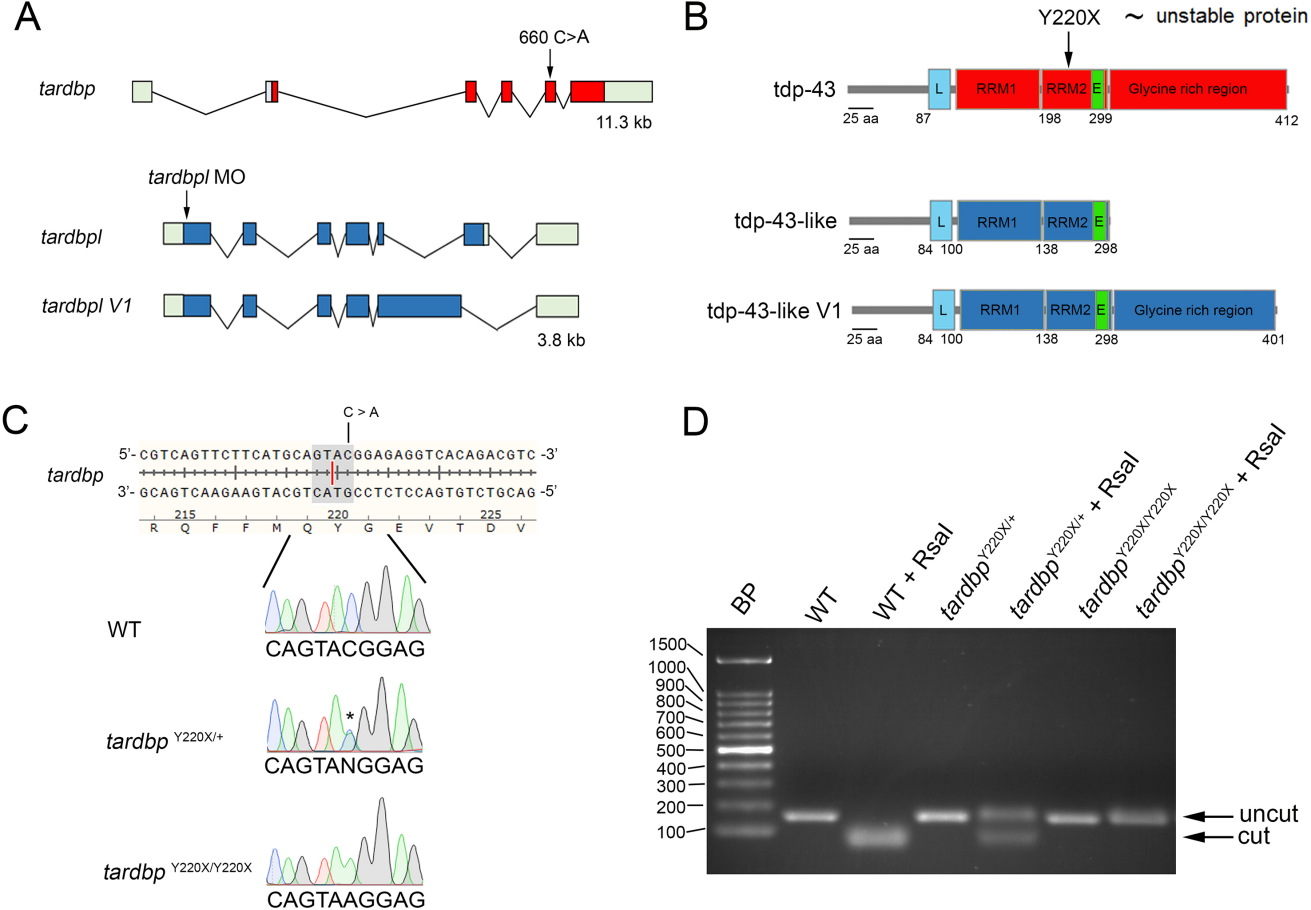
Genomic and protein structures of zebrafish *tardbp* and *tardbpl*. **A,** Arrow in *tardbp* denotes the location of the TILLING-induced mutation (C->A) in exon 5. Arrow in *tardbpl* indicates the ATG morpholino (MO) target site that inhibits translation of both *tardbpl* and *tardbpl V1* transcripts. **B**, The C->A mutation confers a premature stop codon (Y220X) resulting in a truncated and degraded tdp-43 [8]. Loss of tdp-43 is compensated by the increased expression of *tardbpl V1* that contains a c-terminal glycine-rich region. L, nuclear localization sequences; E, nuclear export sequences; RRM, RNA recognition motifs **C**, Example electropherograms of selected sequences encompassing the 660 C->A mutation in WT, heterozygous carriers of the mutation encoding the Y200X missense mutation (*tardbp* ^Y220X/+^) and homozygous larvae (*tardbp* ^Y220X/Y220X^). **D**, Zebrafish carrying this mutation were identified based upon loss of RsaI restriction enzyme binding and cleavage (shaded sequence and red line in C). Undigested or uncut amplicon size was 168 base pairs in length. RsaI-digested amplicons were 97 and 71 base pairs in length.

To further our understanding of synaptic defects that could arise as a result of nuclear clearance of TDP-43 we took advantage of an existing zebrafish loss-of-function model [8]. Unlike the mouse *Tardbp* knockout models that die *in utero* [9–11] zebrafish *tardbp/tardbpl* knockout models survive for over a week [12] and develop externally, facilitating experimental investigations. Zebrafish possess two orthologs of *TARDBP*, *tardbp* (encoding tdp-43) and *tardbpl* (encoding tdp-43-like), though both are expressed throughout development. The first gene, *tardbp*, produces a 412 amino acid protein similar to TDP-43 whereas the second gene, *tardbpl*, generates a truncated tdp-43-like protein of only 303 amino acids lacking the c-terminal glycine-rich domain (Fig 1A and 1B). Inactivation of *tardbp* in itself is insufficient to confer significant abnormalities as loss of normal expression of *tardbp* can be compensated for by an increase in the expression of an alternative splice variant encoded by the *tardbpl* gene, whereby a c-terminal domain similar to the domain encoded by *tardbp* is retained in the *tardbpl* transcript (Fig 1A and 1B). Loss of both *tardbp* and *tardbpl* expression results in early development defects characterized by reduced survival, locomotor deficits and abnormal morphology of motor neuron axon projections in the spinal cord [8, 12]. Building on these findings we examined if synaptic defects arose at the NMJ following tdp-43 depletion as furthering our understanding of synaptic defects will aid in the development of therapeutics to treat ALS.

## Materials and methods

### Zebrafish

Wild-type zebrafish (*Danio rerio)* were bred and maintained according to standard procedures [13]. All experiments were performed in compliance with the guidelines of the Canadian Council for Animal Care and conducted at the Centre de recherche du rentre hospitalier de l’Université de Montréal (CRCHUM) and approved by the Comité institutionnel de protection des animaux of CRCHUM (approval # N15018PMDz). All experiments were performed on sexually undifferentiated zebrafish larvae between 1–11 days post-fertilization (dpf). Humane endpoints were in place during the study and all animals were monitored and assessed daily for well-being as per guidelines established by the Canadian Council of Animal Care committee at the CRCHUM. Behavioral signs of poor health in adult animals necessitating euthanasia included an inability to feed and swim. Physical abnormalities were also monitored daily and adult animals displaying a distended abdomen, skin ulcerations/wounds and skeletal deformities were euthanasia immediately.

### Restriction fragment length polymorphism assay

Genomic DNA was extracted from individual 48 hours post fertilization (48 hpf) larvae using the REDExtract-N-AMP Tissue PCR kit (Sigma-Aldrich) and used as a template for PCR using the following primer sets (*tardbp*-forward CAAGGTATAGATGAACCAATGAGGA) and (*tardbp-*reverse: GTCATCTGCAAAGGTGACAAAAG).

An amplified PCR product of 168 nucleotides was digested with RsaI resulting in two bands (97 and 71 nucleotides respectively) in wild type (WT) larvae. The RsaI restriction site is lost in zebrafish carrying the 660 (C->A) point mutation (Fig 1C), facilitating genotyping of larvae with the Y220X missense mutation (Fig 1D).

### Preparation and injection of *tardbpl* antisense morpholino (MO)

An antisense MO with the following sequence: CCACACGAATATAGCACTCCGTCAT (Gene Tools, OR, USA) was designed complimentary to the region of translational initiation of the *tardbpl* gene (ATGACGGAGTGCTATATTCGTGTGG) in order to inhibit tdp-43-like protein translation, as previously described [8]. The injection of a standard control MO (CoMo) was used with the following sequence: CCTCTTACCTCAGTTACAATTTATA. Microinjections in the 1–2 cell stage embryo were performed as previously described [8]. The injection concentration was optimized by dose-dependent MO toxicity and MOs were injected at a final working concentration of 100 μM to minimize morpholino-induced developmental delay and to yield a consistent phenotype. The MO stock solution was diluted in distilled water with 0.01% Fast Green (Sigma) to a final concentration 100 μM and backfilled in a pulled (Sutter Instrument) thin-walled borosilicate capillary tube and pressure injected into the embryo using a PicoSpritzer III (General Valve). The volume of the injected MO was 5–8 nl. This MO concentration and volume are within the range of a previous report where *tardbpl* gene expression was significantly decreased following the injection of the MO [8].

### Survival and gross morphological analysis

The number of dead or deformed embryos or hatched larvae were counted for the following six treatment groups every day: WT, WT injected with MO, *tardbp*^Y220X/-^, *tardbp*^Y220X/Y220X^, *tardbp*^Y220X/Y220X^ injected with CoMo, *tardbp*^Y220X/Y220X^ injected with MO. At 2 dpf, deformed embryos or larvae were categorized into three groups of deformities: inflated pericardium, abnormal body curvature, a combination of both inflated pericardium and abnormal body curvature. We also examined larval length and eye diameter to examine subtle defects that may have arisen in our treatment groups.

### Locomotor behaviour

Touch-evoked swim escape response was tested at 52 hpf. Larvae were place in the center of a circular aquatic arena (150 mm diameter) and touched lightly on the tails with forceps. The water temperature was maintained at 28.5°C. Locomotor behaviour was recorded digitally at 30 Hz for 10 s (Grasshopper 2 camera; Point Gray Research). Swim duration, swim distance, and maximum swim velocity were quantified off-line using the manual tracking plug-in for ImageJ.

### Whole-cell voltage-clamp recordings in fast-twitch muscle fibers

As previously described [14], zebrafish were anaesthetized in 0.02% tricaine (Sigma) dissolved in modified Evans solution containing the following (in mM): 134 NaCl, 2.9 KCl, 2.1 CaCl_2_, 1.2 MgCl_2_, 10 HEPES, and 10 glucose, adjusted to 290 mOsm, pH 7.8. The zebrafish were then pinned with fine (0.001 inch) tungsten wires through their notochords to a Sylgard-lined dish. The outer layer of skin was removed using a fine glass electrode and forceps, exposing the musculature. The preparation was visualized by oblique illumination (Olympus BX51WI). Standard whole-cell voltage-clamp recordings were obtained from fast-twitch (embryonic white) muscle cells [14]. In these recordings, 1 μM tetrodotoxin (TTX) was perfused over the preparation to inhibit the generation of action potentials and record spontaneous (quantal) miniature endplate currents (mEPC). Glass electrodes (8–12 MΩ) were pulled from filament-containing thin-walled borosilicate glass capillary (A-M Systems) and filled with the following intracellular solution containing (in mM): 116 K-gluconate, 16 KCl, 2 MgCl_2_, 10 HEPES, and 10 EGTA adjusted to pH 7.2, 290 mOsm. Cells were held near their resting potential at -65 mV and series resistance was < 35 MΩ compensated to 40–60%. All electrophysiological data were sampled at 50 kHz using an Axopatch 200B amplifier (Molecular Devices), digitized using a Digidata 1440A (Molecular Devices) and analyzed off-line using pCLAMP 10.1 software (Molecular Devices). It should be noted that the Clampfit software will occasionally detect events that are due to drift in the recordings and report extremely long mEPC kinetics. Based on previously published work [15, 16], we established that fast events arising from the muscle we recorded from would be categorized as having a decay tau constant that is shorter than 4 ms and that slow events, which arise from electrically coupled, neighbouring muscle fibers would be categorized as having a decay tau constant that is between 4 ms and 50 ms. In addition to recording mEPCs, we also recorded the muscle cell membrane potential (Vm), whole-cell capacitance (Cm), membrane resistance (Rm) and access resistance of the electrode (Ra).

### Immunohistochemistry

For whole-mount immunohistochemistry, animals were fixed in 4% paraformaldehyde overnight at 4°C. After fixation the larvae were rinsed several times (1 hr) with phosphate buffered saline (PBS) and then incubated in PBS containing 1 mg/ml collagenase (25 min) to remove skin. The collagenase was washed off with PBS (1 hr) and the larvae were incubated in PBS with Triton X-100 (PBST) for 30 min. The larvae were then incubated with PBST containing 10μg/ml sulforhodamine-conjugated α-bungarotoxin (αBTX; 30 min), which binds irreversibly to acetylcholine receptors (AChRs). The larvae were then rinsed several times with PBST (30 min) and then incubated in fresh block solution prepared from PBS containing goat serum, bovine serum albumin, dimethyl sulfide (DMSO) and Triton X-100, for 1 hr at room temperature and then treated with a solution containing a primary antibody against pre-synaptic synaptotagmin 1 (ZNP-1) (1:100; Molecular Probes) overnight at 4°C. Samples were then washed in PBST and were incubated in block solution containing a secondary antibody (Alexa Fluor 488, 1:1000; Invitrogen) for 6 hr at 4°C. Before imaging, larvae were transferred to a solution containing 70% glycerol and mounted the following day on a slide. The NMJs were visualized using a Quorum Technologies spinning disk confocal microscope with a CSU10B (Yokogawa) spinning head mounted on an Olympus BX61W1 fluorescence microscope and connected to a Hamamatsu ORCA-ER camera. Images were acquired using Volocity software (Improvision) and analyzed using Imaris software (Bitplane).

### Statistical analysis

GraphPad Prism 6 was used to assess data groupings for significance. Statistical analyses used one-way unpaired ANOVA, followed by a *post hoc* Tukey multiple-comparison test. For datasets with a non-normal distribution, nonparametric tests were used where an unpaired Kruskal–Wallis test was performed, followed by Dunn’s *post hoc* test for multiple comparisons. Significance was assessed at *p* < 0.05. N is the number of datasets examined and n is the total number of larvae used in each treatment group. Data are presented as mean ± SEM.

## Results

### Loss of tdp-43/tdp-43-like expression leads to developmental defects and reduced survival

Previous research utilizing zebrafish has demonstrated that loss of normal expression of *tardbp* can be compensated for by an increase in the expression of an alternative splice variant encoded by the *tardbpl* gene (*tardbp* paralog), whereby a c-terminal domain similar to the domain encoded by *tardbp* is retained in the *tardbpl* transcript [8, 12]. Using the ENU-derived mutant zebrafish line containing a single nucleotide substitution (c.660 C > T) resulting in a premature stop codon (TAC -> TAA) at the 220 amino acid of *tardbp* and an unstable protein (Fig 1A and 1B); we first sought to recapitulated the phenotype described by Hewamadduma and colleagues (2013) by injecting an ATG morpholino (MO) targeting *tardbpl* transcript into 1–2 cell stage *tardbp*^Y220X/Y220X^ embryos.

**Fig 2:**
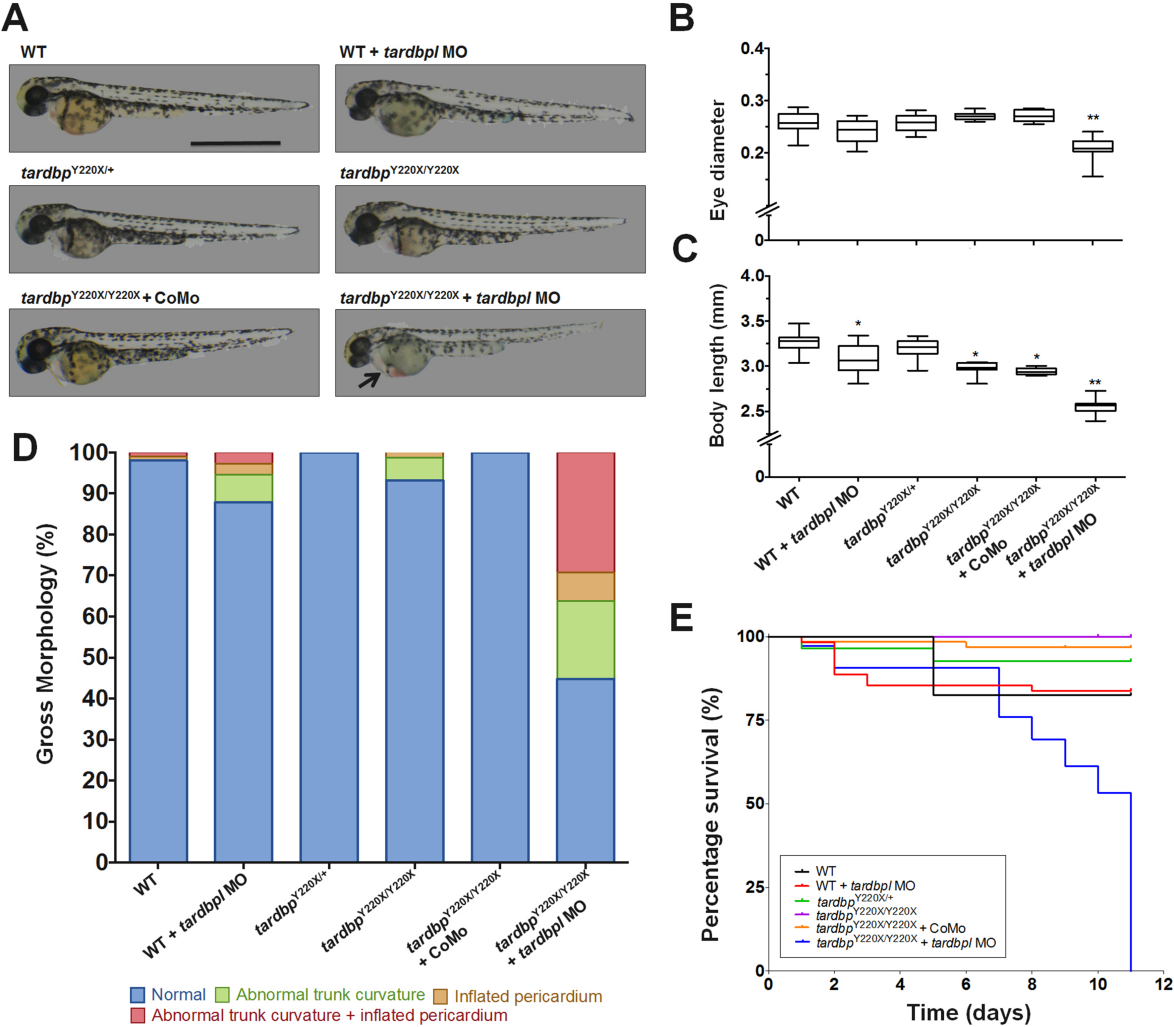
*tardbp*^Y220X/Y220X^ zebrafish embryos injected with an ATG *tardbpl* morpholino (MO) displayed significant morphological defects and reduced survival. **A,** Representative images of 2 dpf larvae from the following treatment groups: WT, WT + MO, *tardbp*^Y220X/+^, *tardbp*^Y220X/Y220X^, *tardbp*^Y220X/Y220X^ + Control MO (CoMo), *tardbp*^Y220X/Y220X^ + MO. Scale bar represents 1 mm, arrow indicates cardiac defect in the *tardbp*^Y220X/Y220X^ + MO treated larva. **B,** Eye diameter and **C,** body length was determined for each treatment group. *tardbp*^Y220X/Y220X^ + MO 2dpf larvae displayed significantly reduced eye diameter compared to all other treatment groups (*p* < 0.01). WT + MO, *tardbp*^Y220X/Y220X^ and *tardbp*^Y220X/Y220X^ + CoMo larvae displayed significantly reduced body length compared to WT larvae (*p* < 0.05) and *tardbp*^Y220X/Y220X^ + MO displayed significantly reduced body length compared to all treatment groups (*p* < 0.01). **D,** Quantification of gross morphological defects observed in all treatment groups indicating a higher incidence of defects in the *tardbp*^Y220X/Y220X^ + MO condition. **E,** Percentage survival curves of all treatment groups tracked over 11 days. *tardbp*^Y220X/Y220X^ + MO-treated larvae displayed reduced survival, with all animals dying by 10 dpf (*p* < 0.0001). N = 2, n = 50, *p < 0.05; **p < 0.01.

Consistent with previous findings, *tardbp*^Y220X/Y220X^ embryos treated with *tardbpl* MO developed abnormally (Fig 2A). Specifically, we observed that *tardbp*^Y220X/Y220X^ larvae treated with the MO displayed a smaller eye diameter compared to all other treatment groups (*p* < 0.01, Kruskal–Wallis test, post-hoc Dunn’s test; Fig 2B). These animals were also found to have a shorter body length compared to all other treatment groups (*p* < 0.01, Kruskal–Wallis test, post-hoc Dunn’s test; Fig 2C). However, WT larvae treated with the *tardbpl* MO also resulted in a small, but significant reduction in body length (*p* < 0.05, Kruskal–Wallis test, post-hoc Dunn’s test; Fig 2C). Additionally, we observed that without MO treatment, or treatment with the standard control MO (CoMo), *tardbp*^Y220X/Y220X^ larvae were shorter when compared to WT larvae (*p* < 0.05, Kruskal–Wallis test, post-hoc Dunn’s test; Fig 2C). These results suggest that either loss of *tardbp* expression or knockdown of *tardbpl* can impact normal trunk development, which is exacerbated when both are coincident. More generally, we also observed a higher incidence of atypical morphological defects such as abnormal trunk curvature, inflated pericardium, or a combination of both in *tardbp*^Y220X/Y220X^ larvae treated with the *tardbpl* MO (Fig 2D). In addition to the developmental abnormalities observed in *tardbp*^Y220X/Y220X^ larvae treated with the *tardbpl* MO, we also assessed changes in mortality and observed a significant decrease in survival compared to all other treatment groups (*p* < 0.0001, Logrank test; Fig 2E) with many animals succumbing by 7 dpf and no larvae surviving beyond 11 dpf. These data confirm a previous report utilizing this mutant line in combination with a *tardbpl* MO (Hewamadduma *et al*., 2013) as well as a double *tardbp*^-/-^; *tardbpl*^-/-^ mutant line (Schimd *et al*., 2013) displaying early mortality.

### Loss of tdp-43/tdp-43-like expression impairs locomotor behaviour

**Fig 3:**
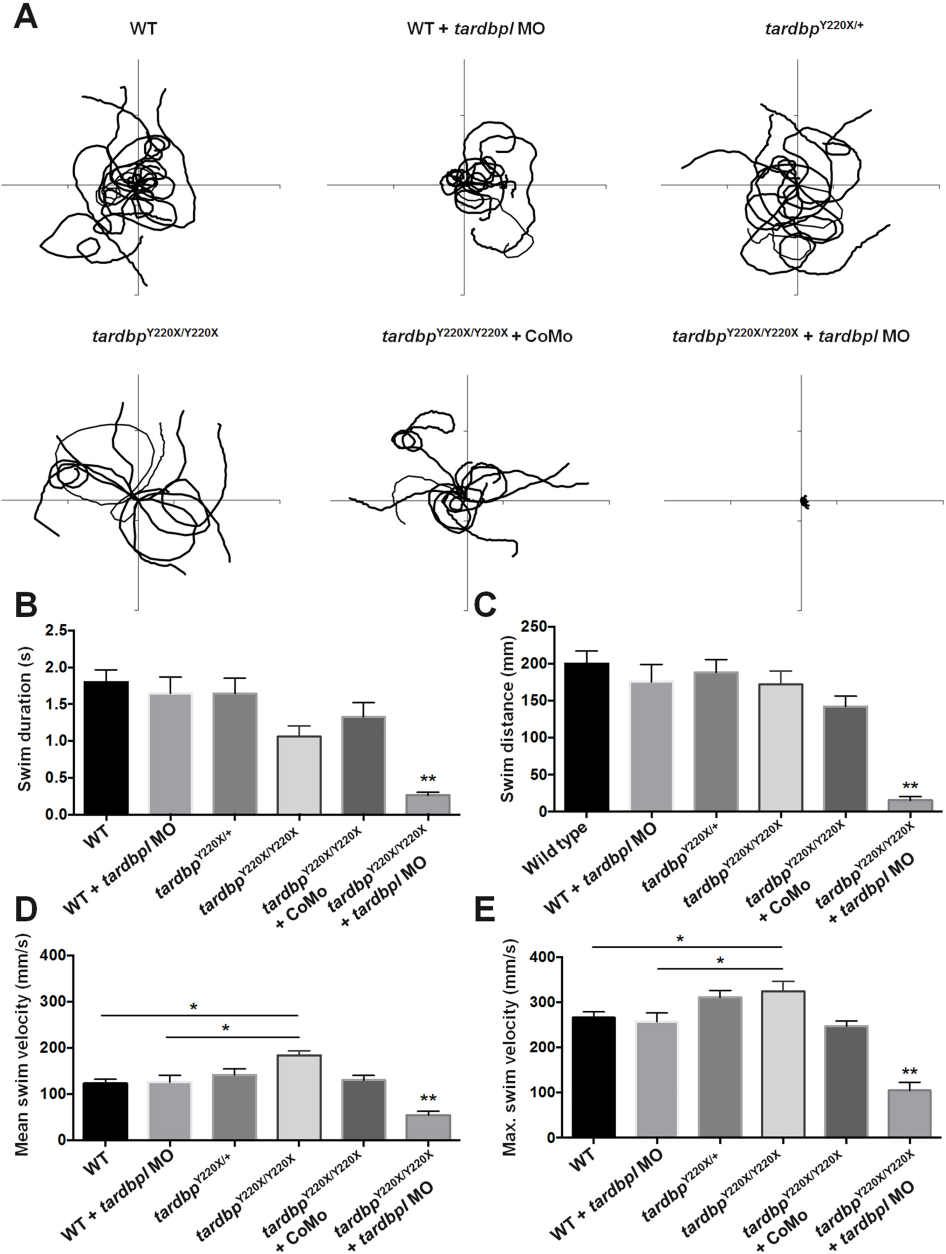
*tardbp*^Y220X/Y220X^ embryos injected with the *tardbpl*-MO displayed severely impaired locomotor behaviour. **A,** 10 superimposed locomotor path traces from each treatment group. Swim duration **B,** swim distance **C,** mean swim velocity **D,** and maximum swim velocity **E,** were calculated for each treatment group. *tardbp*^Y220X/Y220X^ + MO fish displayed significant impairments in all measures of locomotor performance compared to all treatment groups (*p* < 0.01). N = 2, n = 20. Data expressed as mean ± SEM: *p < 0.05; **p < 0.01.

To quantify changes in locomotor behaviour, we performed high-speed video analyses of touch-evoked swim escape responses in 2 dpf larvae. *tardbp*^Y220X/Y220X^ larvae treated with the *tardbpl* MO displayed a dramatic impairment in locomotor behaviour compared to all other treatment groups (Fig 3A). More specifically, these larvae displayed a significant reduction in mean swim duration (*p* < 0.01, one-way ANOVA, post-hoc Tukey test; Fig 3B) mean swim distance (*p* < 0.01, one-way ANOVA, post-hoc Tukey test; Fig 3C), mean swim velocity (*p* < 0.01, one-way ANOVA, post-hoc Tukey test; Fig 3D) and maximum swim velocity (*p* < 0.01, one-way ANOVA, post-hoc Tukey test; Fig 3E) compared to all other treatment groups. These results are consistent with the severe deficit in locomotor function characterized previously [8, 12]. Unexpectedly we also observed a slight but significant increase in the mean and maximum swim velocity of *tardbp*^Y200X/Y220X^ larvae when compared to WT and WT zebrafish treated with the *tardbpl* MO (*p* < 0.05, one-way ANOVA, post-hoc Tukey test; Fig 3D and 3E). However, we did not observe the same finding when we treated these animals with the standard CoMo, suggesting that microinjection of embryos may in itself have a slight effect on these two parameters of locomotor performance.

### Loss of tdp-43/tdp-43-like expression leads to aberrant synaptic activity at the NMJ

Previous work has established that trunk musculature in larvae lacking both *tardbp* and *tardbpl* display severe defects in muscle patterning characterized by disordered and smaller myofibrils poorly separated from one another (Schmid et al 2013). To further our understanding of the defects arising in trunk musculature we performed whole-cell patch clamp recordings of fast twitch muscle cells to examine physiological abnormalities arising in larval *tardbp*^Y220X/Y220X^ zebrafish treated with the *tardbpl* MO. Specifically, we examined the electrophysiological properties of fast-twitch muscle fibers in 2 dpf zebrafish larvae as these are similar to the muscle fibers affected by the disease in patients with ALS [17, 18]. Fast-twitch muscle cells in WT and *tardbp*^Y220X/Y220X^ larvae displayed no significant differences in passive membrane properties between each other (Table 1). We did observe a significant reduction in membrane resistance (Rm) in WT larvae treated with the *tardbpl* MO when compared to untreated WT larvae (*p <* 0.05, one-way ANOVA, post-hoc Tukey test). No differences were observed in membrane potential (Vm) and membrane resistance (Rm) in *tardbp*^Y220X/Y220X^ larvae treated with the *tardbpl* MO when compared to WT larvae, but we did observe an increase in membrane capacitance (Cm) in these animals when compared to all treatment groups (*p <* 0.01, one-way ANOVA, post-hoc Tukey test). This suggests that loss of tdp-43 and tdp-43-like V1 impairs the normal electrical decoupling of fast-twitch muscles cells during development.

**Table 1:** Properties of fast-twitch muscle fibers in 2 dpf larval zebrafish.

	Vm (mV)	Cm (pF)	Rm (MΩ)
WT	-68.5 ± 1.8 (12)	22.76 ± 2.42 (12)	39.77 ± 5.22 (12)
WT + *tardbpl* MO	-68.99 ± 2.16 (12)	21.08 ± 2.94 (12)	20.14 ± 2.25 (12)*
*tardbp*^*Y220X/Y220X*^	-69.33 ± 1.1 (15)	22.73 ± 2.2 (15)	30.69 ± 3.32 (15)
*tardbp*^*Y220X/Y220X*^ + *tardbpl* MO	-66.29 ± 2.68 (7)	47.73 ± 14.39 (7)**	33.79 ± 5.87 (7)

One-way ANOVA, post-hoc Tukey test. Asteriks represent statistical significance compared to WT larvae.

*p < 0.05

**p < 0.01.

Numbers in parentheses represent sample sizes.

**Fig 4:**
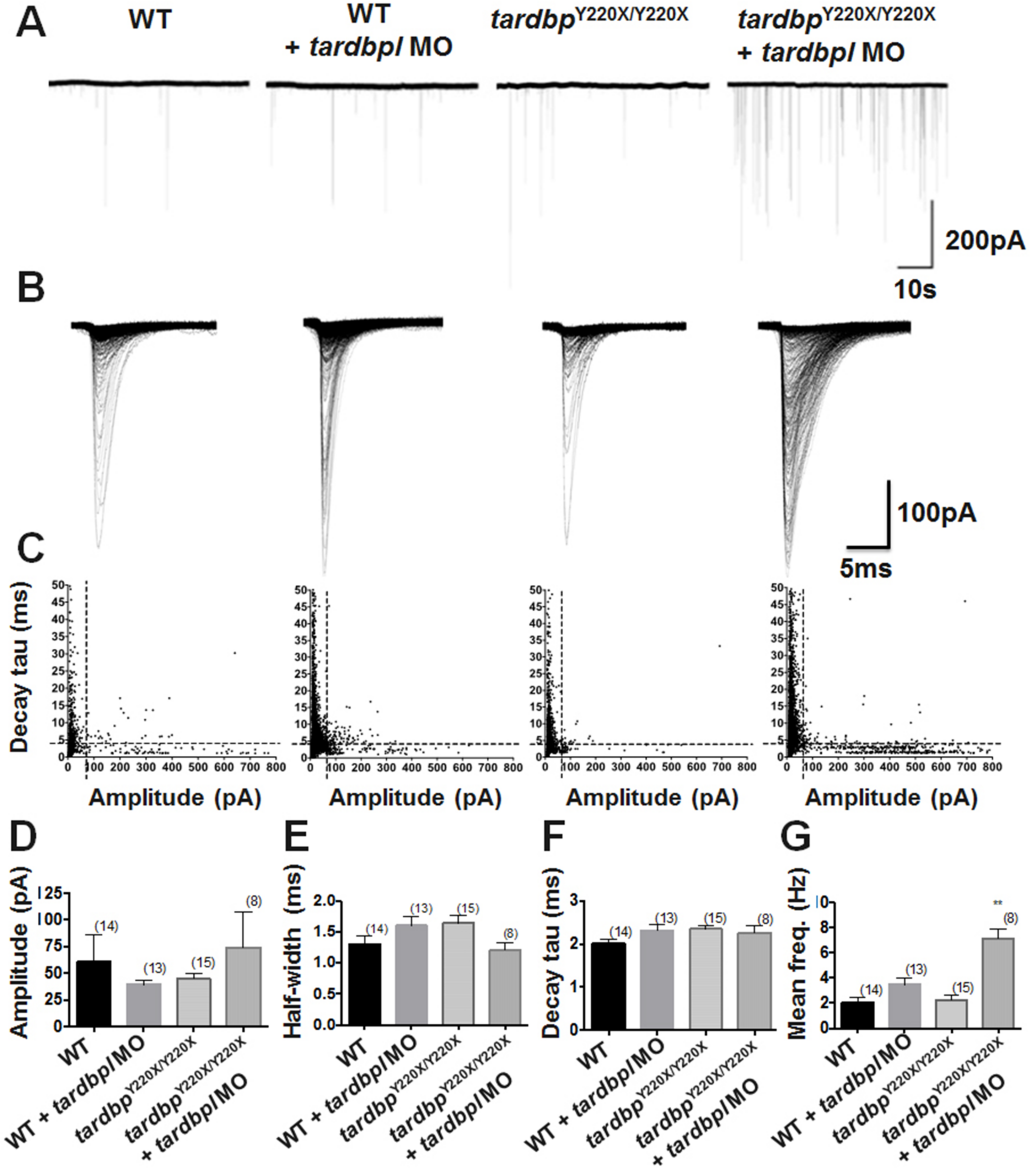
Whole-cell voltage-clamp recordings of *tardbp*^Y220X/Y220X^ + MO larvae displayed a higher frequency of mEPC events at 2 dpf. **A,** 60 second sample traces from 10 minute recordings from the following 4 treatment groups: WT, WT + MO, *tardbp*^Y220X/Y220X^ and *tardbp*^Y220X/Y220X^ + MO. **B,** Overlay of all mEPC events recorded during a 10 minute recording in each treatment group. **C,** Scatterplots graphing exponential decay constant (*tau*) *vs* amplitude distributions for 5 individual recordings from each treatment group. Plotted mEPCs can be divided into two populations; slow decay *tau* (*tau* > 4 ms), activity detected from neighbouring muscle fibers, and fast decay *tau* (*tau* < 4 ms), arising from the recorded muscle itself. Black dotted lines indicate the separation of these event types. Amplitude (pA) **D,** half-width (ms) **E,** decay *tau* (ms) **F,** and mean instantaneous frequency (Hz) **G,** were measured for mEPCs in each treatment group. Only fast mEPCs (*tau* < 4 ms) arising from the recorded muscle were analyzed. *tardbp*^Y220X/Y220X^ + MO larvae displayed a significantly higher mean instantaneous frequency compared to all other treatment groups (*p* < 0.01). No other significant differences were observed. Numbers in parentheses represent sample sizes. Data expressed as mean ± SEM: *p < 0.05; **p < 0.01.

Previous research from our laboratory utilizing a human TDP-43 over-expression model has revealed defects in quantal release at the NMJ in zebrafish expressing mutant human *TARDBP*^G348C^ but not in larvae expressing human *TARDBP*^WT^ [19]. It remains to be determined what synaptic defects arise following loss of tdp-43/tdp-43-like expression. To address this, we recorded miniature end-plate currents (mEPCs) in the following treatment groups: WT, WT treated with *tardbpl* MO, *tardbp*^Y220X/Y220X^ and *tardbp*^Y220X/Y220X^ larvae treated with *tardbpl* MO (Fig 4A and 4B), and then plotted all detected events by their decay tau constant (*tau*) and mEPC amplitude. mEPCs displaying slower kinetic properties have been previously demonstrated to arise in neighbouring muscle cells and are filtered electrically through gap junctions [15, 20]. As expected, whole-cell voltage clamp recordings revealed a range of mEPCs varying in amplitude and decay constant (Fig 4B) that could be separated into either fast (*tau* < 4 ms) or slow (*tau* > 4 ms) events (Fig 4C).

Homozygous *tardbp*^Y220X/Y220X^ larvae treated with the *tardbpl* MO were found to have a higher number of overall mEPCs, comprised of both fast and slow mEPC events (Fig 4A and 4B; *p* < 0.01, Kruskal-Wallis, Post-hoc Dunn’s multiple comparison test). To determine if the increased number of total mEPCs events observed in this treatment group was driven by a particular population of events we compared the proportion of fast and slow events to the total number of mEPCs in each treatment group and found no significant differences among our treatment groups. This suggests that the increased number of quantal events recorded in fast twitch muscle cells from homozygous *tardbp*^Y220X/Y220X^ larvae treated with the *tardbpl* MO were not driven by a single population of mEPCs, but rather occurred irrespective of a particular decay constant.

After establishing that there was an equal increase in the number of slow and fast mEPC events, we went on to assess changes in the characteristics of fast mEPC events only, as these are the mEPCs that presumably arise in the recorded muscle itself. We did not find any differences in mean amplitude of mEPCs (Fig 4D), mean half-width duration (Fig 4E), nor mean decay constant (Fig 4F). However, we did observe a significant increase in the mean instantaneous frequency of mEPCs in homozygous *tardbp*^Y220X/Y220X^ larvae treated with the *tardbpl* MO when compared to all other treatment groups (*p* < 0.01, one-way ANOVA, post-hoc Tukey test; Fig 4G). This suggests that the combined loss of tdp-43 and knockdown of tdp-43-like expression results in either a gain of presynaptic active zone area or an augmentation of the molecular mechanisms that enable quantal vesicular release at the NMJ.

### Abnormal motor neuron branching and synapse formation at the NMJ following the loss of tdp-43/tdp-43-like expression

**Fig 5:**
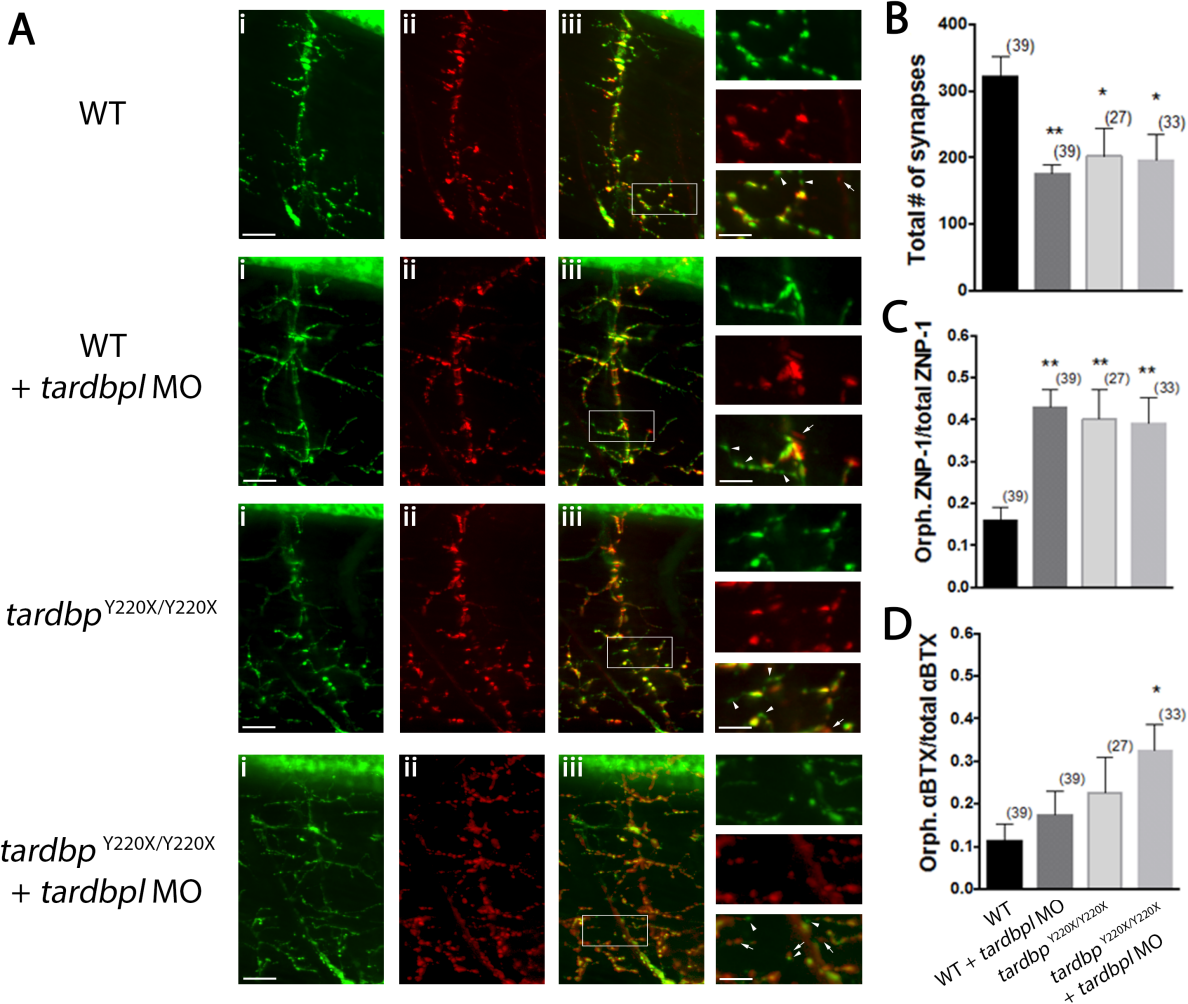
Motor neuron projections in *tardbp*^Y220X/Y220X^ + MO larvae show increased number of orphaned presynaptic and postsynaptic puncta. **A**, Representative images of single ventral root projection double-labelled for ZNP-1 (presynaptic marker, i) and sulforhodamine-conjugated αBTX (postsynaptic marker, ii). WT larvae display extensive co-localization of both ZNP-1 and αBTX (merged image, iii). Scale bar in (iii) and insets represent 25 μm and 10 μm respectively, arrowheads indicate orphaned ZNP-1 puncta and arrows indicate orphaned αBTX labelling. **B**, Quantification of the number synapses formed, quantified as the number of colocalized ZNP-1 and αBTX puncta. WT + MO (*p* < 0.05), *tardbp*^Y220X/Y220X^ (*p* < 0.01) and *tardbp*^Y220X/Y220X^ + MO larvae (*p* < 0.01) all had a significantly reduced number of synapses at the NMJ compared to WT larvae. **C**, Quantification of orphaned presynaptic ZNP-1 puncta over total number of ZNP-1 puncta. WT + MO, *tardbp*^Y220X/Y220X^ and *tardbp*^Y220X/Y220X^ + MO larvae displayed significantly higher proportion of orphaned ZNP-1 puncta compared to WT animals (*p* < 0.01). **D,** Quantification of orphaned postsynaptic αBTX staining over total number of αBTX puncta. *tardbp*^Y220X/Y220X^ + MO fish displayed significantly higher proportion of orphaned αBTX puncta compared to WT larvae (*p* < 0.05). Numbers in parentheses represent the number of somites analyzed for each treatment group. Data expressed as mean ± SEM: *p < 0.05; **p < 0.01.

Prior research has demonstrated that knockdown of *tardbpl* in *tardbp*^Y220X/Y220X^ larvae results in a higher number of motor neuron axon defects [8]. As our observations of synaptic function indicated aberrant, increased connectivity at the NMJ, we decided to examine pre- and post-synaptic structural components by performing whole mount immunohistochemistry of the NMJ (Fig 5A). To visualize presynaptic components of the NMJ we incubated the fixed tissue with ZNP-1, and counter stained with sulforhodamine-conjugated α-bungarotoxin (α-BTX) to visualize acetylcholine receptor clusters postsynaptically. We observed that WT larvae treated with *tardbpl* MO, *tardbp*^Y220X/Y220X^ larvae and *tardbp*^Y220X/Y220X^ treated with *tardbpl* MO all displayed a reduction in the number of synapses at the NMJ (colocalization of ZNP-1 and α-BTX) compared to WT animals (*p* < 0.05, one-way ANOVA, post-hoc Tukey test; Fig 5C). Furthermore, we found that WT larvae treated with *tardbpl* MO, *tardbp*^Y220X/Y220X^ larvae and *tardbp*^Y220X/Y220X^ treated with *tardbpl* MO all had higher proportions of orphaned ZNP-1 puncta (absence of colocalization of α-BTX) over total ZNP-1 puncta when compared to WT animals (*p* < 0.01, one-way ANOVA, post-hoc Tukey test; Fig 5D). Additionally, we observed that *tardbp*^Y220X/Y220X^ larvae treated with *tardbpl* MO displayed a higher proportion of orphaned α-BTX receptor staining (absence of colocalization of ZNP-1) over total α-BTX receptor staining when compared to WT group (*p* < 0.05, Kruskal-Wallis, Post-hoc Dunn’s multiple comparison test; Fig 5E). These latter two observations confirm that in addition to synaptic defects, the loss of either tdp-43 or tdp-43-like leads to defects in the formation/maintenance of presynaptic components of the NMJ and when lost in combination both pre- and post-synaptic components of the NMJ are destabilized.

## Discussion

Advances in our understanding of TDP-43’s cellular role in synaptic function have been limited. This may in part be due to difficulties in generating viable vertebrate and invertebrate loss-of-function models of TDP-43. Existing TDP-43 knockout models [9–11] display early embryonic lethality and die *in utero* between days 3.5 and 8.5 of embryonic development. In *Drosophila melanogaster*, *TBPH*^*-/-*^ (ortholog of *TARDBP*) mutants die at the second instar larval stage [21], precluding physiological examinations in loss-of-function models both later in development and in adulthood. Here, we build upon a previously described TILLING mutant containing a premature stop codon (Y220X) in *tardbp*, resulting in an unstable and degraded tdp-43 protein [8]. Loss of tdp-43 expression has been shown to be compensated by the upregulation of a novel *tardbpl* transcript variant and early mortality was only seen following knockdown of *tardbpl* expression in *tardbp*^Y220X/Y220X^ mutants [8]. In this study we extended our understanding of the physiological consequences following the loss of expression of both zebrafish *TARDBP* orthologs and demonstrated that in addition to reduced survival and impairments in locomotor behaviour, synaptic defects arose at the NMJ. This was observed as an unexpected increase in the frequency of quantal release of acetylcholine synaptic vesicles at the vertebrate NMJ.

Though it was not the sole aim of this research, we did confirm many previous findings utilizing the *tardbp*^Y220X/Y220X^/*tardbpl* MO model [8] as well as confirmed observations in a double *tardbp* and *tardbpl* knockout model [12]. We observed that *tardbp*^Y220X/Y220X^ zebrafish survived to sexual maturity and swam normally but at 48 hpf we observed a slight but significant reduction in *tardbp*^Y220X/Y220X^ larval body length when compared to WT animals (Fig 2). Treatment with the *tardbpl* MO in WT animals also resulted shorter larval length but severe developmental defects only arose in *tardbp*^Y220X/Y220X^ larvae following knockdown of *tardbpl* expression (Fig 2). It is therefore likely that basal expression of *tardbp* and *tardbpl* play a role in zebrafish development. In addition, abnormalities in the vascular system of larval zebrafish lacking both tdp-43 and tdp-43-like have been observed [12]. Though we did not examine vasculature defects in detail, we did observe that a significant proportion of *tardbp*^Y220X/Y220X^ larvae treated with the *tardbpl* MO displayed an inflated pericardium (Fig 2), and consistent with previous findings [8], *tardbp*^Y220X/Y220X^ zebrafish larvae treated with the *tardbpl* MO died prematurely with no animals surviving beyond 11 dpf (Fig 2).

As we were interested in furthering our understanding of physiological defects that arise in *tardbp*^Y220X/Y220X^ larvae with knockdown expression of *tardbpl*, we characterized locomotor function, to provide insight into where defects in the motor system occur. Using previously developed methods to assess locomotor function in zebrafish larvae [19], we found that the majority of the animals were unable to swim in response to a touch (Fig 3). Though these experiments determined that there is an overall defect in locomotor ability, it was not clear whether defects were occurring in the muscular and/or nervous systems. A key advantage of the larval zebrafish preparation is that it lends itself easily to physiological examinations of the muscular system.

Using the patch-clamp technique, we recorded passive membrane properties of fast-twitch muscles of the zebrafish trunk. Homozygous *tardbp*^Y220X/Y220X^ larvae injected with the *tardbpl* MO displayed an increased membrane capacitance, suggesting incomplete decoupling of gap junctions at embryonic stages of muscle development. Though we did not examine hemichannel expression patterns, it is conceivable that the combined loss of *tardbp* and *tardbpl* expression perturbs gap junction assembly/disassembly. Evidence for altered gap junctions expression patterns have been noted in an SOD1 mouse model, where at symptomatic stages both spinal cord astrocytic and oligodendrocytic connexin expression patterns have been shown to be profoundly affected and have been proposed to facilitate neuronal death [22]. Following depletion of brain Tdp-43 using a morpholino-based strategy in mice, yielded several hundred genes that displayed altered expression patterns, one of which was the upregulation of *Gje1* expression, encoding the hemichannel Connexin 29 (Cx29) [3]. Cx29 is normally localized to the small myelin sheath inner membrane in oligodendrocytes [23] and expression of Cx29 has been found to be increased in the penumbra following traumatic brain injury [24]. Though one must be mindful of inappropriate comparison between healing following traumatic brain injury and ALS, similarities between how damage and diseased tissue cope with their respective challenges may exist, and the expression of gap junctions may be a common feature. What role gap junctions play in the nervous and muscular systems of TDP-43-related ALS cases has yet to be investigated, but could be an interesting avenue for future studies.

Though the majority of our measures of passive membrane properties of fast-twitch muscle cells did not differ among our treatment groups, we did observe changes in quantal release of acetylcholine at the NMJ in *tardbp*^Y220X/Y220X^ mutants treated with the *tardbpl* MO. Previous assessments of quantal release at the NMJ in a *TARDBP* zebrafish model [25] revealed that expression of mutant human *TARDBP*^G348C^, but not *TARDBP*^WT^, was associated with a reduction in spontaneous release of Ach at the NMJ [19]. We were therefore surprised to observe a significant increase (3.5 times larger than WT) in the frequency of spontaneous release of synaptic vesicles in zebrafish with depleted tdp-43/tdp-43-like (Fig 4). This change suggests a presynaptic defect that could result from additional endplates or enhanced Ach release at the presynaptic side of the NMJ. To examine the former possibility we performed immunohistochemistry on the NMJ to investigate structural changes that could account for increased quantal vesicle release. We found that *tardbp*^Y220X/Y220X^ larvae injected with *tardbpl* MO displayed an increase in both the number of orphaned presynaptic (ZNP-1) and postsynaptic (αBTX) puncta compared to WT (Fig 5). Furthermore, WT larvae injected with *tardbpl* MO, *tardbp*^Y220X/Y220X^ larvae and *tardbp*^Y220X/Y220X^ larvae injected with *tardbpl* MO all displayed a decrease in the number of synapses formed compared to WT animals (Fig 5). The presence of fewer synapses as well as evidence of significant axonal projection defects and orphaned Ach receptors in *tardbp*^Y220X/Y220X^ larvae injected with *tardbpl* MO suggests that the increase in frequency of spontaneous release of synaptic vesicles at the NMJ is likely not a result of supernumerary endplates, but resulting from another unknown mechanism. One potential mechanism could involve a homeostatic compensation by motor neurons in an effort to increase the strength of impaired NMJs. This phenomenon was brought to the forefront in *Drosophila* NMJ studies where impaired cellular depolarization of muscle cells following expression of the inward rectifying potassium channel Kir2.1 [26] or genetic manipulations of the muscle-specific glutamate receptor function [27] can be compensated by an increase in neurotransmitter release presynaptically. It is conceivable that a similar mechanism could be arising in our tdp-43 loss of function model and represents an interesting avenue for future studies.

Work addressing NMJ deficits in the aforementioned *TARDBP* zebrafish overexpression model observed activity-dependent presynaptic impairments where motor neurons expressing *TARDBP*^G348C^ were more excitable than those expressing *TARDBP*^WT^ in that they generated higher frequencies of evoked action potentials following current injection [19]. Similarly, in an *SOD1* zebrafish model, larvae expressing mutant *SOD1* were also found to display presynaptic deficits characterized by increased neuronal stress in glycinergic interneurons and reduced glycinergic transmission to motor neurons [28]. Though we only examined defects at the NMJ it is possible that these deficits may also arise upstream in the neural circuits that coordinates locomotor behaviour.

In this study we extended our understanding of the synaptic and structural defects that arise at the NMJ in a vertebrate loss-of-function model of TDP-43. To our surprise, we observed augmented quantal release of synaptic vesicles despite a reduction in the number of synapses at the NMJ. Though the exact mechanisms coordinating this phenomenon remain to be determined, it represents a hitherto undocumented presynaptic defect in an ALS model. Future studies using this model to examine the functional connectivity between motor neurons and muscle cells using optogenetic tools will allow us to better understand the synaptic defects that we believe are relevant for furthering our understanding of ALS pathology.
